# Strengthened HPV vaccination among adolescents living with HIV, – Findings of a Pilot QI project in Kampala Metropolitan, Uganda

**DOI:** 10.1371/journal.pone.0356686

**Published:** 2026-08-31

**Authors:** Oliver Ombeva Malande, Munube Deogratias, Julia Jerono Songok, Sekinah Hassan, Varsetile Varster Nkwinika, Andrew Munyalo Musyoki, Doreen Sekibombo, Diana Antonia, Rachel Nakatugga Afaayo, Sam Njunwamukama, Mutunga Nzoka, Sabrina Bakeera Kitaka

**Affiliations:** 1 East Africa Centre for Vaccines and Immunization (ECAVI), Kampala, Uganda; 2 Department of Paediatrics & Child Health, Moi University, Eldoret, Kenya; 3 Department of Paediatrics & Child Health, Makerere University, Kampala, Uganda; 4 Department of Public Health Pharmacy and Management, Sefako Makgatho Health Sciences University, Pretoria, South Africa; 5 Department of Paediatrics and Child Health, Mulago National Referral Hospital, Kampala, Uganda; 6 Department of Virology, Sefako Makgatho Health Sciences University, Pretoria, South Africa; 7 South African Vaccination and Immunisation Centre (SAVIC), Sefako Makgatho Health Sciences University, Pretoria, South Africa; 8 Department of Microbiology, Sefako Makgatho Health Sciences University, Pretoria, South Africa; 9 Mildmay Uganda (MUg), Kampala, Uganda; 10 Joint Clinical Research Centre (JCRC), Kampala, Uganda; IAVI, UNITED STATES OF AMERICA

## Abstract

**Introduction:**

Recent reports from Kampala indicate low uptake and completion of the human papillomavirus (HPV) vaccine among adolescents, especially amongst those living with HIV. However, the drivers of this low uptake are poorly understood. This study aimed to determine the current HPV vaccine coverage; assess the knowledge, attitude and practices influencing HPV vaccine uptake among adolescent girls living with HIV; and pilot an HPV vaccination program while documenting lessons learned and experiences at two urban HIV treatment centres the Joint Clinical Research Centre (JCRC) and Mildmay Uganda in Kampala, Uganda.

**Methods:**

This was a descriptive cross-sectional mixed methods study employing both qualitative and quantitative data collection methods, carried out between 03/12/2024–30/08/2025, among adolescent girls living with HIV in Kampala, Uganda. A total of 371 were enrolled, of which 182 from JCRC and 189 from Mild May Uganda. At enrolment, 11 adolescents declined consent to participate. Quantitative data on knowledge of HPV and HPV vaccination was collected through interviews, with any additional demographic data being obtained from medical records at the treatment centres. Qualitative data included four focused group discussions (FGDs) of adolescent girls and 32 key informant interviews (KIIs) with focal health workers (Paediatricians, nurses, counsellors) at the two centres were conducted.

**Results:**

Overall, 138/371 (37%) of the adolescents reported having heard about the HPV vaccine, while 233/371 (63%) had not. At enrolment, only 51 out of 371 (14%) participants had initiated vaccination. Among those who initiated vaccination, 51% had received only the first dose. By the end of the study, 342/371 (92%) of adolescents had completed the recommended two doses of the HPV vaccine. This study focused on increasing vaccine uptake amongst the HIV positive adolescents at the two centres. Most adolescents with a good level of knowledge about HPV or those attending school reported to have initiated vaccination. Identified barriers to vaccine uptake included inadequate knowledge about HPV and the benefits of vaccination; limited availability of HPV vaccines, and the absence of routine HPV immunisation programs within HIV treatment centres or programs specifically targeting adolescents living with HIV.

**Conclusion:**

In these two urban HIV care and treatment centres, vaccination against HPV among eligible adolescents is way lower than recommended 90 percent. Adolescent girls living with HIV demonstrated low levels of knowledge about the HPV vaccine and poor access to vaccination services, despite being receptive to vaccination. This study showed that HPV vaccine uptake can be substantially improved within HIV care and treatment centers by reducing barriers to HPV vaccination uptake when the vaccines are made readily available and accessible, either in existing HIV care and treatment centres or in the new integrated care centres in areas where government programs have moved to implement HIV care integration into mainstream medical services.

## Background

Vaccine-preventable diseases (VPDs) are a major public health problem in developing countries [1]. Immunisation is a cost-effective intervention for reducing morbidity and mortality associated with VPDs, particularly in high-burden settings [1–3]. Despite global efforts to reduce child mortality, approximately 10 million children under the age of ﬁve die every year, the majority of whom are in low income countries [4,5]. It is estimated that vaccines prevent more than three million child deaths each year, and children who are fully immunised by nine months of age experience lower morbidity and mortality related to VPDs [4]. Immunisation is therefore a central intervention towards attaining Sustainable Development Goal (SDG) 3, which aims to reduce under-five mortality to fewer than 25 deaths per 1000 live births by 2030 [3]. Inequalities and inequities in vaccine coverage persist across populations and are driven by multiple factors, including low educational attainment of parents/caregivers, cultural/religious beliefs, caregiver age, geographical terrain, limited access to health facilities, refugee status, population mobility, negative messaging/anti-vaccine sentiments, and low socioeconomic status of the parents/caregivers [2,6–9].

Human papilloma virus (HPV) infection is typically acquired soon after initiation of sexual activity, with up to 80% of sexually active women acquiring HPV during their lifetime; and approximately 75% of new infections occurring among individuals aged 15–24 years [10]. The HPV is implicated in approximately 4.5% of all cancers worldwide and the high-risk human papillomavirus types cause over 90% of cervical cancers, as well as genital warts and other cancers. [11,12]. It causes nearly all cervical cancers with genotypes 16 and 18 accounting for up to 70% of cases [13]. Other cancers caused by HPV include 95% of anal cancers, 70% of oropharyngeal cancers, 70% of vaginal and vulvar cancers, 60% of penile cancers [14–16]. In addition, HPV causes non-cancerous diseases such as genital warts and recurrent respiratory papillomatosis [16].

Cancer of the cervix is the most common cancer among women living with HIV, who are up to six times more likely to develop the disease compared to their HIV negative counterparts [16]. Adolescent girls living with HIV, even in the context of effective antiretroviral therapy (ART), are at a higher risk of acquiring HPV infection, harboring multiple HPV genotypes, experiencing persistent infection, and progressing more rapidly from cervical dysplasia to invasive cancer than their HIV negative counterparts [17–19]. The HPV vaccine is highly effective for pre-teens and young adults, and reduces cervical cancer risk by up to 90% recipients and provides long-lasting immunity, and has been shown to be safe and effective in people living with HIV (PLWHIV), with enhanced immunogenicity observed among those with controlled HIV viral replication and no overt immunodeficiency [18,20]. The quadrivalent HPV vaccine showed 98% efficacy in the prevention of cervical intraepithelial neoplasm and 100% efficacy against genital warts in HIV negative people and protects against cervical cancer, and strains responsible for anal, oral, vaginal, vulvar, and penile cancers. Among PLWHIV, efficacy ranges from 89% to 100% for each of the 4 genotypes following completion of a three-dose schedule. It is projected that by 2030, cervical cancer will cause more than 443,000 deaths in women per year globally, with the majority occurring in sub-Saharan Africa [21].

Uganda has recently revised its national HPV vaccination guidelines, transitioning from a two-dose schedule (at 0 and 6 months) for girls aged 9–14 years and three-dose schedule (at 0, 2 and 6 months) for girls aged 15 years and older or those who are immunocompromised, including those living with HIV, to a single-dose schedule for HIV-negative girls and a two-dose schedule for girls living with HIV [22,23]. Despite these efforts, HPV vaccination coverage in Uganda remains suboptimal, with up to 78% of the adolescent girls aged 10–14 years not yet vaccinated [24]. Studies conducted in Kampala have also shown low completion rates of the second HPV vaccine dose among adolescents aged 9–15 years [25]. The Uganda National Expanded programme on immunisation (UNEPI) records indicate nationwide HPV second-dose uptake of 65% in the year 2018–2019, 38% in 2019–2020, 56% in 2020–2021, and 56% in 2021–2022 [23]. Recent reports documenting HPV vaccine uptake among adolescents attending the Mulago National Hospital adolescent clinic in Kampala show that 69.8% of girls completed two-doses of the HPV vaccine, while 30.2% received only one dose [25]. The aim of this study was therefore to determine current HPV vaccine coverage; assess the knowledge, attitude and practices affecting HPV vaccine uptake and completion among adolescent girls living with HIV; to increase vaccine uptake amongst the HIV positive adolescents while documenting lessons learned and experiences at the Joint Clinical Research Centre (JCRC) and Mildmay Uganda (MUg).

## Methods

### Study design setting and approval

This was a descriptive cross-sectional mixed methods study employing both qualitative and quantitative data collection methods [26,27], carried out from 03/12/2024–30/08/2025 incorporating both qualitative and quantitative data collection methods, with a follow up quality improvement component. The study was carried out at two HIV care and treatment centres within the Kampala metropolitan area – the Joint Clinical Research Centre (JCRC) and Mildmay Uganda (MUg). These centres manage large cohorts of adolescents living with HIV and were selected in consultation with the Ministry of Health immunisation program (UNEPI), Kampala City Council Authority (KCCA) and Wakiso district immunisation program focal persons. At the time of the study, neither centre had an established routine immunisation service, however, a small number of the adolescents had previously received HPV vaccination at other facilities or through government outreach immunisation activities. The target population comprised all adolescent girls aged 9–19 years living with HIV and attending the care and treatment services at the two centres. Quantitative data on sociodemographic characteristics, viral load, and World Health Organization (WHO) HIV Staging was obtained from medical records of the girls at the treatment centres during the period from 03/12/2024–30/08/2025. Additional quantitative data on knowledge of HPV and HPV vaccination was collected through interviewer-administered questionnaires using a piloted data collection tool. The qualitative data was obtained through four focus group discussions (FGDs) with adolescent girls, each comprising eight participants, giving a total of 32 participants, and 32 Key Informant Interviews (KIIs) of focal health workers. These included heads of the treatment centres, nurses providing adolescent care, counsellors, paediatricians, and immunisation focal persons at district, division, and Ministry of Health levels involved in HPV vaccine roll out. Eligible adolescent girls aged 9–19 years living with HIV and attending the two participating treatment centres were screened consecutively over the study period. The FGDs were carried out after completion of the quantitative interviews and were used to explore themes and topics that had not been exhaustively addressed in the interviews.

### Inclusion and exclusion criteria

We included adolescent girls attending HIV care and treatment services at the two selected centres who were eligible for HPV vaccination and whose caregivers provided consent for them to receive the vaccine. Assent where applicable was also obtained from those adolescents included in the study. Adolescents who had already completed the recommended HPV vaccination series prior to the study were excluded. Additional exclusion criteria included refusal to provide informed consent or inability to recall relevant information required for the study. Girls who had received the first HPV vaccine dose outside the clinic setting were also excluded. For key informant interviews, healthcare providers involved in the delivery of HIV care and adolescent services at the two treatment centres were eligible for inclusion.

### Data collection and analysis

Following ethical approval, the principal investigators (PIs) engaged the KCCA Health Officer and Wakiso District Health Officer (DHO), who introduced the study team to the Assistant District Health Officers (ADHOs) responsible for immunisation under the EPI programme. The PIs and research assistants met with the immunisation focal persons, including cold-chain technicians, to brief them on the study procedures. Subsequently, the PIs were introduced to leadership at JCRC and MUg to discuss study logistics, including questionnaire administration, FGDs, and cold chain arrangements for HPV vaccine provision. Alongside data collection, eligible adolescents who had missed HPV vaccination were offered vaccination through the routine immunisation programme. The goal was to ensure that consenting, previously unvaccinated adolescents received at least one dose of the HPV vaccine and to assess vaccine uptake among adolescent girls living with HIV. Adolescents who declined vaccination received targeted education and counselling; those who continued to decline were interviewed, and their reasons for refusal were documented. Previous HPV vaccination status was verified using vaccination cards and participant history.

The FGD participants were selected through purposive sampling to ensure maximum variation in the sample. The two FGDs were conducted at each treatment centre, yielding a total of four FGDs. Discussions were conducted in either English or Luganda, depending on participant preference, audio recorded, and later transcribed verbatim. Participants in FGDs were different from those who participated in individual interviews. Data saturation on most themes was achieved. Each FGD was facilitated by well- trained research assistants, with one moderator and one note-taker per session. A pre-developed topic guide was used to explore knowledge, perceptions, attitudes, fears, practices, and uptake related to HPV vaccination. The guides were developed a priori based on existing literature and the research team’s professional experience, and appropriate translation and back-translation procedures were applied. Focus group discussions were conducted in private spaces within the treatment centres and lasted between 60 and 80 minutes. Transcripts were prepared using Microsoft Word 2016 by bi-coordinate bilingual transcribers, fluent in both English and local languages. After transcription, the transcripts were imported into the NVivo version 12 program for thematic analysis, allowing identification of both anticipated and emergent themes. Unique study identifiers were used to enable cross-checking of data across sites while maintaining participant anonymity. All data was securely stored in lockable cabinets and password-protected electronic devices.

### Follow up component

The follow-up component of this study involved tracking the adolescents who received the first vaccine dose for 6 months from the first dose to ensure completion of the second and/or third dose. Health workers at both centres received twice-weekly supportive supervision and mentorship from the PIs and study team, focusing on HPV vaccination concepts and service delivery. Additionally, six hybrid monthly webinars were conducted across the two study sites targeting all healthcare workers. Topics included: Immunology and types of Vaccines; The HPV vaccines, scheduling, types and dosing; Adverse events following immunization; Cold chain, record keeping, Vaccine storage, transport and waste management; Ministry of health Policy Shift to single dose for HIV-uninfected adolescents and two-dose schedules for adolescents living with HIV; and Vaccine Hesitancy, myths and misconceptions related to HPV vaccination.

### Ethical considerations

Ethical approval to carry out the study was obtained from the Makerere University School of Health Sciences ethics committee for MAKSHREC-2024–733 for the period 04/09/2024–04/09/2025, conducted in the period 03/12/2024–30/08/2025 as per guidelines under the Declaration of Helsinki. Separate individual written informed consent was also obtained from each study participant interviewed individually and in the FGDs and from parents/caregivers of all minors and for all those interviewed in the KIIs was obtained for the study, for the interviews, recording, transcription, dissemination of all proceeds from the study, and publishing. In situations where additional information was required which was not covered by any of these levels of consent described above, particularly extraction from patients’ records and additional secondary data and carried out during the period from 03/12/2024–30/08/2025, a consent waiver was sought from or waived/granted by the Makerere University School of Health Sciences ethics committee for MAKSHREC-2024–733. All the proceeds of FGDs and KIIs were kept confidential. All data were coded and were only accessible to the research team. No participant had any of their identity revealed, and every participant was assigned a unique record identifier, and that all reports, future publications, and records of proceeds of this study do not and shall not in any way contain any potentially identifying participant information. All the data and all the information collected during this study is safely stored. Any additional findings not in this manuscript is included in the supporting information files provided.

## Results

### Quantitative component findings

#### Study profile.

**Fig 1:**
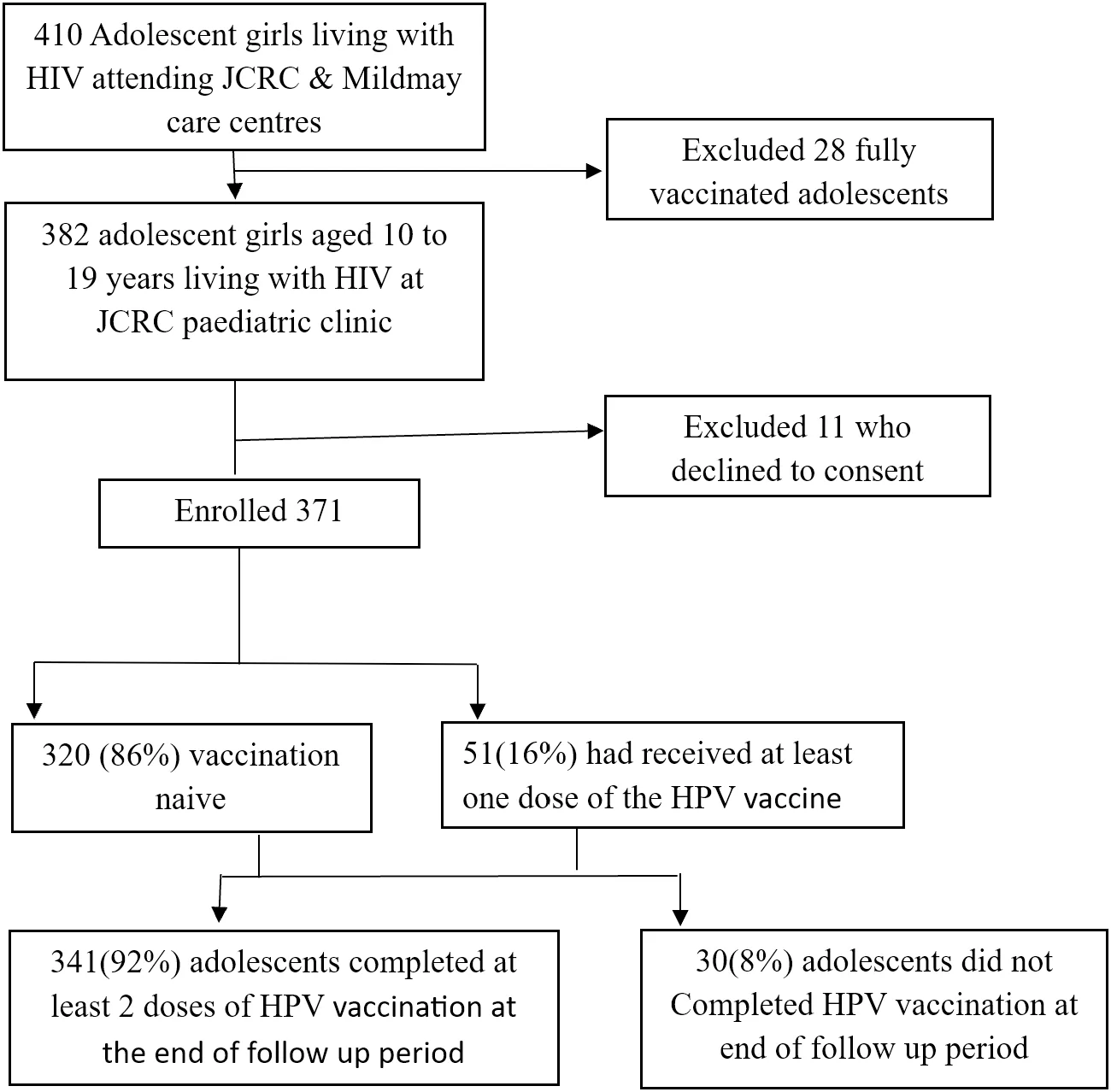
Study profile showing participant recruitment at JCRC and Mildmay Uganda.

Between the period between 03/12/2024 and 30/08/2025, a total of 410 adolescent girls living with HIV were screened at the JCRC and Mildmay Uganda HIV care and treatment centers. Of these, 28 adolescents who had already completed HPV vaccination and 11 who declined consent were excluded, resulting in a final study sample of 371 participants. This included 182 adolescents at JCRC and 189 at Mildmay Uganda. By the end of the study, 342/371 (92%) adolescents had completed their HPV vaccination, while 29/371 (8%) had received only one dose of the vaccine. Additional participant characteristics are shown in Table 1 and Fig 1.

**Table 1:** Baseline characteristics of 371 HIV positive adolescent girls included in the study.

Variables	Frequency N = 371	Percentage (%)
**Treatment Centre**		
JCRC	182	49.1
Mildmay	189	50.9
**Age of Adolescents**		
9-14	117	32
15-19	254	68
**Religion**		
Catholic	185	49.9
Protestant	121	32.6
Muslim	35	9.4
Others	30	8.1
**Child’s caregiver**		
Both parents	234	63
Single parent	82	22
Other*	55	15
**Do you know about the Human Papilloma Virus**		
No	276	74.4
Yes	95	25.6
**WHO HIV staging**		
Stage I	89	24.0
Stage II	187	50.5
Stage III	75	20.0
Stage IV	20	5.5
**Participants Perceptions on Causes of Cervical Cancer**		
Other uncommon beliefs	115	31
Infections/STDs	28	7.5
Unprotected sex/sexual behavior	25	6.7
HPV or virus	17	4.5
Misconceptions (abortion, smoking, candida etc.)	11	2.9
Poor hygiene	8	2.1
Not sure	7	1.9
Family planning misconceptions	3	0.8
Self-medication or medication misuse	3	0.8
Early sexual activity/pregnancy	1	0.3
I don’t know	133	35.8

*Includes: Grandparents, aunt, other guardians.

#### Human papillomavirus knowledge.

The study found that among the 371 adolescent girls living with HIV, only 95/371(25.6%) reported being aware of the human papillomavirus (HPV), indicating generally low levels of awareness in this population. Further, the 95 participants who reported awareness of HPV were further interviewed regarding specific aspects of HPV, and their responses are summarized in Table 2.

**Table 2:** HPV Knowledge among 95 adolescent girls living with HIV.

Responses from those who knew about HPV	Frequency N = 95	Percentage (%)
**HPV is a virus**		
No	19	20
Yes	76	80
**HPV is sexually transmitted**		
No	37	39
Yes	58	61
**HPV can cause cervical cancer**		
No	16	16.8
Yes	79	83.2
**There’s a relationship between HPV and HIV**		
No	44	46.3
Yes	51	53.7
**HPV occurs in girls alone**		
No	19	20
Yes	76	80

#### Initiation of HPV vaccination at the start of the study.

Among the 371 who participated in the study, 320 (86.3%) had not initiated HPV Vaccination at baseline, while only 51 (13.7%) had initiated vaccination. The majority of participants who had not initiated HPV vaccination reported that they were either not aware about the vaccine or they did not know that they needed to be vaccinated. Other reasons are shown in the notes on Table 3. Among the 13% of respondents that had initiated but not completed the HPV vaccination schedule, the common reasons they cited why adolescents don’t initiate vaccination were: lack of awareness regarding of the need for subsequent doses, lack of the vaccine at the health facility when they returned, and failure by healthcare workers to offer follow-up doses. Other reasons are detailed in the notes on Table 3.

**Table 3:** Initiation of HPV vaccination among the 371 adolescents at the start of the study.

Have you started HPV vaccination	Frequency N = 371	Percentage (%)
No*	320	86.3
Yes**	51	13.7

*Reasons given by respondents for not having started HPV Vaccine

1. *I didn’t know that I needed to*

2. *I didn’t find it safe*

3. *No one offered it to me*

4. *I wasn’t aware about the vaccine*

5. *I didn’t think it works*

6. *My health facility didn’t have the vaccine*

7. *My caregiver discouraged me from it*

8. *The vaccine is painful*

9. *The vaccine is harmful*

10. *Passed age limit by then; the nurse refused her at school; I didn’t know how to get it*

** Reasons for having initiated but never completed the schedule

1. *It is not yet the time I was asked to come for next dose*

2. *7% Lack of awareness (didn’t know needed to, wasn’t aware, how to get it)*

3. *Vaccine not available at health facility*

4. *No one offered it to me*

5. *Passed age limit by the time of next dose*

6. *Nurse refused her at school*

### Qualitative component findings

The qualitative analysis included 4 focus group discussions involving adolescents and 32 key informant interviews (KIIs) with facility-based health workers, EPI programme and immunization focal persons. The data were analysed thematically using a hybrid inductive–deductive approach. First, transcripts were read repeatedly for familiarisation. Initial codes were then developed deductively from the study objectives and key review concerns (knowledge, attitudes, access, integration into HIV care, completion, and service improvement), while also allowing inductive coding for unexpected issues emerging from participants’ accounts. Related codes were compared across FGDs and KIIs, grouped into candidate categories, refined iteratively, and consolidated into final themes. Illustrative quotations were then selected to represent the range of views across participant groups, including less common but analytically important perspectives. This analytic process yielded seven major themes: (1) fragmented knowledge and persistent misconceptions about HPV, cervical cancer, and the vaccine; (2) fear, rumours, and adverse-event narratives undermining confidence in vaccination; (3) Vaccine uptake decisions were socially mediated by parents, peers, schools, and health workers; (4) HPV vaccination was weakly integrated into routine HIV care; (5) access, documentation, and follow-up barriers limited initiation and completion; (6) participants proposed practical service improvements to increase uptake and completion and (7) Challenges with adherence and follow up in care centres and other health system barriers.

#### Theme 1: Fragmented knowledge and persistent misconceptions about HPV, cervical cancer, and the vaccine.

Across adolescent, caregiver, and health worker accounts, awareness of HPV and HPV vaccination was present but often shallow, fragmented, or mixed with incorrect beliefs. Adolescents and caregivers frequently recognised HPV as being linked to cervical cancer, but they also described inaccurate routes of transmission, uncertainty about eligibility, confusion about where the vaccine is administered, and incomplete understanding of dose schedules. Health workers similarly noted that the system had often emphasised screening more than vaccination, leaving both families and some providers with incomplete knowledge.

*“Some of them have never even heard about the HPV Vaccine.” (Adolescent participant, FGD, JCRC)*

*I think that if an infected person uses a dirty toilet or latrine, and then another person uses it, that person can also get infected with the HPV virus.” (Adolescent participant, FGD, JCRC)*

*“Growing up, it was linked to girls who had not yet become sexually active, so some people still think that once a girl has had sex, she cannot get the vaccine.” (Volunteer medical worker, KII, JCRC)*

*“It is not that HPV is completely new here; it is just not talked about enough. People do not know enough about it to have a proper conversation.” (Volunteer medical worker, KII, Mildmay UG)*

#### Theme II: Fear, rumours, and adverse-event narratives undermined confidence in vaccination.

A second theme concerned fear and mistrust. Adolescents described being discouraged by warnings that the vaccine was painful or dangerous. Participants and health workers reported widespread rumours that HPV vaccination causes infertility, death, or other bodily harm. These narratives were not peripheral; they were repeatedly described as active barriers that shaped refusal, delay, or hesitancy. Uncommon but influential adverse-event stories, including rashes or dizziness after vaccination, also circulated and appeared to reinforce mistrust.

*“Some people mislead others. You may be going for vaccination, and someone tells you, ‘If you take it, you will die.’” (Adolescent participant, FGD, Mildmay)*

*“One of the things that is actually killing uptake is awareness, because there is a lot of misinformation and disinformation out there.” (Vaccine technical officer, KII, Mildmay)*

*“Why was it that some of us felt dizzy after vaccination?” (Adolescent participant, FGD, JCRC)*

*“After my sister received the vaccine, she developed a rash, and I became very scared and feared to get the vaccine.” (Adolescent participant, FGD, JCRC)*

*“There are some people who say these vaccines will make the girls fail to give birth or become barren, and once the parent is convinced of that, the adolescent also refuses.” (Village health team member, KII, Mildmay)*

#### Theme III: Vaccine uptake decisions were socially mediated by parents, peers, schools, and health workers.

The decision to start or complete HPV vaccination was rarely described as an individual choice alone. Instead, it was embedded in a social environment shaped by parents, caregivers, peers, teachers, schools, and providers. Adolescents reported hearing about HPV through schools, hospitals, friends, and conversations in the community. They said that parents remained decisive gatekeepers, especially where girls were young or where consent was expected. Health workers also described peers, teachers, and school structures as important facilitators of uptake and completion.

*“Once the parent is not comfortable with the vaccine, the child will also not take it.”(Vaccine technical officer, national programme KII)*

*“There were some girls, my friends, I was explaining to about HPV vaccine, but they had many questions and I gave up.” (Adolescent participant, FGD, JCRC)*

*“The caregivers always tell us that it is important to inform parents and guardians when their children are going to be vaccinated, because many times parents are not informed.” (Nurse, KII, JCRC)*

*“The main factor is the parent. If the parent does not consent and says, ‘You are not vaccinating my child,’ then that child cannot be vaccinated.” (Enrolled nurse, KII, Mildmay)*

#### Theme IV: HPV vaccination was weakly integrated into routine HIV care.

Health worker informant interviews revealed that HPV vaccination was often not embedded consistently within routine HIV care pathways. In several settings, providers described counselling or referral as the main approach rather than vaccination within the HIV care and treatment centres. Some respondents explicitly stated that HPV vaccine had not been fronted as a standard service within adolescent HIV clinics. Others described fragmented arrangements in which HPV vaccine information was given in one unit but vaccination was offered elsewhere. This lack of integration appears to have contributed to missed opportunities, weak ownership within HIV clinics, and inconsistent follow-through. This approach left some health workers within HIV treatment centres lacking adequate information or up to date knowledge on HPV vaccine.

*“Most times we are focused on the routine review and solving immediate complaints, but for HPV specifically there has not been a lot of enthusiasm.” (Paediatric clinician, KII, JCRC)*

*“Many health workers actually do not know the schedule. Even personally, I know the vaccine is there, but the details of when and how long are not always clear.” (Paediatric clinician, KII, JCRC)*

*“It is discouraging when people come to the hospital and do not find the vaccine available. They go back and tell others that the service is not there.” (Community mobilizer at JCRC, KII)*

*“From my experience, HPV is not usually presented as one of the routine services within adolescent HIV clinics. Yet if it were included, it would be very beneficial because these girls already come there for care.” (Public health officer, national-level KII)*

#### Theme V: Access, documentation, and follow-up barriers limited initiation and completion.

Participants consistently described structural barriers that affected both uptake and completion. These included distance to health facilities, missed opportunities for vaccination, stock-out of vaccines, weak communication between schools and families, incomplete use of vaccination cards and registers, and inconsistent follow-up for subsequent doses. Several health workers described practical systems for cards, registers, and phone calls, but these systems were not uniformly strong across sites. Provider interviews also revealed uneven familiarity with evolving HPV vaccine dose schedules, which suggests that service delivery systems were changing faster than frontline understanding in some settings.

*“Some people are deep in the village and want the vaccine, but transport to the hospital is a problem, so they remain there.” (Community worker, KII, Mildmay)*

*“Some girls forget their appointment dates, some lose their cards, and some receive vaccines from school without proper documentation, so follow-up becomes difficult.” (Adolescent nurse, KII, JCRC)*

*“There are times when we have stock-outs of HPV vaccine, and that completely disorganizes uptake.” (Vaccine technical officer, national programme KII)*

*“Usually, uptake of the second dose is poor because some children have already left for school so uptake of the second dose is really low.” (Vaccine technical officer, national programme KII)*

*“If vaccination is done on the same date as their clinic appointment, it is easier. But if the days are separated, it is not easy for them.” (Young adolescent peer supporter, KII, Mildmay)*

*“We have a register where each vaccinated girl is recorded, including whether she received the second dose. We also give them cards.” (Midwife, KII, Mildmay)*

#### Theme VI: Participants proposed practical service improvements to increase uptake and completion.

The final theme concerned solutions. Across FGDs and KIIs, participants suggested more intensive community and school sensitisation, stronger counselling, better communication with parents, reliable vaccine availability, closer integration with HIV clinics, use of peers and VHTs, and more deliberate follow-up systems. Importantly, respondents did not describe adolescents living with HIV as fundamentally resistant to vaccination. Rather, they saw poor uptake as a modifiable systems problem that could improve if information, access, and follow-up were strengthened.

*“I request you go to school and educate girls on the danger of the disease and vaccinate them, then next time you go back and vaccinate them and also educate them as a whole.” (Adolescent participant, FGD, Mildmay)*

*“We need to educate health workers too not everyone is confident talking about HPV, the schedule, or where girls should go.” (Enrolled nurse, KII, Mildmay)*

*“The most important thing would be sensitizing parents telling them the importance of the vaccine, the dangers of the disease, and why early protection matters.” (Enrolled nurse, KII, Mildmay)*

*“What we can do is to go to schools… then also go to communities to educate them… because parents are big decision-makers in the lives of these children.” (Health worker, Mildmay KII)*

*“Working with religious leaders to address the misconceptions and then use of radio talk shows and community outreaches to continue educating the masses about the importance of vaccination… by doing so we shall have high uptake.” (Programme supervisor, Kampala KII)*

#### Theme VII: Challenges with adherence and follow up in care centres and other health system barriers.

The health workers reported challenges related to adherence to the HPV vaccination schedule and follow-up of adolescents living with HIV. Some adolescents receive vaccines through school-based programmes, resulting in fragmented records and difficulties in tracking vaccination status. In addition, the absence of formal immunisation services within HIV care and treatment centres complicated follow-up and continuity of care, making it difficult to ensure timely completion of the vaccination schedule.:

*“Some of the girls forget their appointment dates and it’s really hard to follow up, some of them lose their cards and it’s really hard to follow up, some of them get vaccines from schools and they are not given cards and it’s really hard to follow up, that’s some of the reasons.” (KII, Adolescent Nurse).*

*“Most times we have a challenge like following them up most time they can do first dose second dose, most times you need to call them and some still don’t come.” (KII, Nursing officer).*

*“There are issues with follow up, where these children get lost from the clinic, when they are lost it is just like the experience with ARVs, some also disappear or go away from the clinic and you have to look for them so it may delay treatment rather the receipt of vaccine The other one is school, sometimes they go to school and they don’t get a chance to have an outreach.” (KII, Health Worker, Public Health Officer, JCRC).*

*“For me, for the second dose, we went to the hospital and they told us that the vaccines were over. We went to a different hospital and they said there was no vaccine.” (FGD participant, JCRC).*

*“Availability of the vaccines is still a challenge because in most cases when you ask a clinician where one can access the HPV vaccine; it will take him time to think of where one can get it. Most clinicians are not aware of where to refer patients if need arises unlike hepatitis B which is available at every facility either government or private.” (KII, Health worker JCRC).*

*“I struggled to get the vaccine for my daughter; we received the first dose and for second dose we were referred to a facility without the vaccine.” (KII, Mild May).*

#### Cross-cutting uncommon/minor themes.

In addition to the dominant themes, several less frequent but important findings include the belief that HPV can be acquired from dirty toilets, shared clothes, or contaminated family-planning procedures; uncertainty about whether boys and men should also be vaccinated; concern that outreach-delivered vaccines are less trustworthy than hospital-based vaccines; and a view among some health workers that adolescents living with HIV sometimes avoid added vaccination because they already feel burdened by lifelong treatment.

*“Seeing they are on ARVs, they just see that their lives are going to be messed up with by adding these vaccines.”* (Health worker, Mildmay KII)

#### Concerns regarding adverse events following HPV vaccination.

Some respondents expressed fears regarding adverse events following HPV vaccination, particularly concerns about future fertility:

*“I went with my parent to get the first dose though it was painful, and as a result, I have never gone for the second dose.” (Adolescent at JCRC FGD).*

#### Negative Peer influence.

Peer influence played a role in vaccine refusal among some adolescents:

*“There are many friends at school who don’t want the vaccine, and advice other girls not to get it. Some says the vaccine causes cancer or kills you slowly.” (FGD participant, JCRC).*

#### Negative attitude of health workers.

Some of the adolescents described unfriendly or discouraging interactions with health:

*“There is a nurse who is very harsh, and shouts at us or talks in a mean way and doesn’t encourage you when you have fears or questions about vaccines.” (FGD participant, Mildmay).*

## Discussion

### Quantitative component: Uptake and completion of HPV vaccine in adolescent girls living with HIV

At baseline, HPV vaccine uptake among adolescent girls living with HIV was extremely low at 14%, far below Uganda’s national target of 80% coverage in girls aged 10–14 years, and the WHO 2030 cervical cancer elimination target of 90% full vaccination by age 15 years [28]. Among the 371 who participated in the study, 320 (86.3%) had not initiated HPV Vaccination at baseline, while only 51 (13.7%) had initiated vaccination. This highlights a substantial gap in vaccine uptake, suggesting limited access to vaccination services, inadequate awareness of HPV and its benefits, or other structural barriers that need to be addressed through targeted health education and service delivery improvements. These findings underscore the need to better understand the reasons for low uptake, which was a primary objective of this study. Although data on HPV vaccination among adolescents living with HIV in Africa is limited, this low uptake raises serious concerns regarding the effectiveness of parallel HIV care and treatment programmes in integrating routine immunisation services. The parallel model of care in comprehensive care clinics and donor funded programs lends itself to the possibility of routine government immunisation and other disease prevention/health promotion failing to reach vulnerable HIV-infected populations.

Globally, HPV vaccination coverage was estimated to be at 12.2% in 2018 [29], comparable to the baseline uptake in this study. However, experiences from South Africa demonstrate that high uptake and completion rates are achievable [30]. This study in the KwaZulu-Natal Province of South Africa, reported uptake rates exceeding 97% across all three doses and WHO-UNICEF cumulative data indicate that by 2020, 61% of South African girls aged 15 years had completed the HPV vaccine series [30]. In the current study, focused follow-up and service integration resulted in substantial improvements: dose one uptake among targeted adolescents increased to 97%, and completion of the second dose reached 92%. The remaining 8% were largely untraceable due to disruptions caused by changes in U.S. government funding models, which resulted in some adolescents being transferred to government public facilities closer to their homes, thus dropping out of the HIV care and treatment centres they belonged to. The ripple effect of this shift by the US government in funding priorities and approaches will be felt for many years to come [31,32].

This study found that low uptake of HPV vaccine is not driven by a single problem such as lack of awareness alone, but by the interaction of demand-side concerns and service-delivery weaknesses.

The awareness about HPV and HPV vaccination was partial, inaccurate, or operationally insufficient. Adolescents and caregivers frequently recognised that HPV is linked to cervical cancer, yet that awareness coexisted with misconceptions about transmission, uncertainty about eligibility, confusion about where the vaccine is administered, and incomplete understanding of dose schedules. In Oyam District, northern Uganda, Rujumba and colleagues found that girls, caregivers, teachers, community health workers, and health workers described inadequate information, uncertainty about the vaccination schedule, and weak communication as key barriers to both initiation and completion [33]. As has been reported earlier, inadequate information about HPV infection and HPV vaccine benefits, unclear communication from health workers, and concerns about vaccine safety contributed to poor timely completion of the second dose among girls attending Mulago adolescent clinic [25]. A school-based mixed-methods study from Kampala also found that knowledge gaps, negative perceptions, and persistent parental concerns remained major constraints even in an urban setting [34].

What is notable in the present study is that these knowledge gaps were not limited to adolescents and caregivers. Some health workers also described uncertainty about schedules, eligibility boundaries, and how HPV vaccination should fit within HIV care. That shifts the problem from community ignorance alone to a service-readiness issue. Even when girls living with HIV are already engaged in care, opportunities for vaccination can still be missed if providers are not confident enough to counsel, document vaccination status, and guide girls through referral or follow-up. This is particularly important because WHO continues to prioritise HPV vaccination for immunocompromised individuals and people living with HIV, recommending a minimum of two doses and, where feasible, three doses for those known to be HIV infected or otherwise immunocompromised [35]. In that context, poor provider clarity about schedules and pathways can directly undermine uptake among the very group that policy seeks to prioritise.

In our earlier study conducted at the Mulago Hospital adolescent clinic in Uganda among girls aged 9–14 years [25], we found that 30.2% girls had received only one dose of the HPV vaccine, while 69.8% girls had completed the two recommended doses. That study included both HIV-infected and uninfected girls. It however revealed that when vaccination programmes are structured and deliberate in their follow-up of eligible girls, uptake and completion rates improve significantly [36]. This highlights the need for focused and intentional programming in efforts aimed at improving HPV vaccine uptake [36]. Similarly, a district-based study among adolescent girls aged 13–19 years attending secondary schools in Wakiso District of Uganda, showed a low HPV vaccination uptake rate of 9.2% [37], a clear indicator that barriers to uptake of the HPV vaccines are widespread across the country [24]. In Lira District, a study among adolescent girls aged 12–17 years showed that of 49.6% had not received the HPV vaccine, 18.0% had received one dose, and 14.8% had received two does [38]. Furthermore, a recent unpublished study by Sekinah et al. [39] among HIV-infected adolescents found that 74.36% had not initiated HPV vaccination and only 1.5% had completed all the three recommended doses. It is hoped that the recent policy shift to a one-dose schedule for HIV-uninfected adolescents and a two-dose schedule for HIV-infected adolescents will go a long way toward improving HPV vaccine uptake and completion rates [25,37,40].

### Qualitative component of the study

#### Barriers to HPV vaccine uptake in adolescent girls living with HIV.

We documented several barriers hindering HPV vaccine uptake among HIV-infected adolescents in Kampala, Uganda. These included the lack of clear immunisation programmes specifically targeting this vulnerable population, with HIV treatment centres operating parallel to mainstream public health programmes and thus often being excluded from routine child survival initiatives. Other barriers included limited knowledge about the HPV vaccine among caregivers and eligible adolescents; myths and misconceptions; fears of adverse events following immunisation (AEFIs); health system deficiencies; loss to follow-up and inadequate supply of vaccines in some seasons. Numerous studies across Africa have documented inadequate knowledge/information about HPV, cervical cancer and the role of HPV vaccination in cancer prevention as a significant barrier to uptake [25,33,36]. Improving vaccination coverage among adolescents who are unaware of the disease or the vaccine remains challenging. There is therefore a need for the Ministry of Health and the national immunisation programme to fully integrate HIV care and treatment centres into routine immunisation services. The fear of AEFIs remains a major hindrance to HPV vaccine uptake, not only in this study but in other reports as well [33]. Other barriers reported in other studies include inadequate numbers of healthcare workers to deliver vaccination services at the health facility [41], myths, rumors and misconceptions about the HPV vaccine, including fears of infertility or barrenness [33,41]; parental refusal to consent for vaccination [33]; and school non-attendance or dropout, which reduces HPV vaccination completion rates because they are likely to miss school-based vaccination days [33].

This study found that fear and mistrust were not peripheral issues but central mechanisms shaping hesitancy. Participants described fears of pain, infertility, death, and bodily harm, and these concerns were reinforced by rumours, anecdotal adverse-event stories, and broader distrust of vaccination. These concerns are highly consistent with prior Ugandan studies. In northern Uganda, fear of side effects, discouragement from peers and caregivers, and negative community messages were reported as major reasons girls failed to start or complete the HPV vaccine series [33]. In Kampala, Patrick et al. similarly identified concerns about efficacy, safety, and misinformation as barriers to timely completion [25]. The Kampala school-based study by Bitariho et al. also reported negative parental beliefs, superstitions, and safety concerns around HPV vaccination, indicating that fear and misinformation are not confined to rural or remote settings [34].

What appears more specific in our study is the way these fears interacted with the lived experience of HIV care. Some respondents suggested that adolescents already taking lifelong ART, or other medicines, could view HPV vaccination as one more burden. Others described the added injection as being interpreted by parents through the lens of vulnerability, reproductive threat, or over-medication. This layering of HPV vaccine hesitancy onto the realities of long-term HIV treatment is important because it suggests that generic vaccine messaging may be insufficient for girls living with HIV. A more recent Ugandan study from Greater Masaka similarly found low HPV vaccine uptake among girls and young women living with HIV, and reported that stronger HPV knowledge was associated with higher odds of vaccination [42]. Our findings deepen that result by showing why knowledge matters: it is not merely factual understanding, but explanation strong enough to counter fear, fertility rumours, and treatment fatigue.

Another finding was that vaccine decisions were socially mediated rather than individually made. Adolescents described learning about HPV through schools, peers, hospitals, and community conversations, but caregivers repeatedly positioned themselves as the most trusted and decisive source of final advice. This point directly addresses the reviewer query about trusted sources of advice. In our data, parents and caregivers were the central gatekeepers for younger girls, while peers, teachers, schools, and providers shaped whether girls felt informed and supported enough to act. This aligns with wider Ugandan evidence. Rujumba et al. found that caregiver approval, teacher involvement, and community-based mobilization strongly influenced HPV vaccination decisions in northern Uganda [33]. In Mulago, Patrick et al. showed that peer influence and healthcare worker recommendation positively influenced timely completion [25]. In western Uganda, Asiimwe et al. reported that caregiver education and knowledge of HPV vaccination were significantly associated with uptake, again reinforcing the importance of the household informational environment [43].

Our study adds to this literature by showing that social mediation was not only about consent, but also about coordination. Parents wanted advance notice from schools, adolescents described trying to persuade peers, and some providers described peer supporters and home visits as practical routes for follow-up. In other words, trusted advice operated through a layered network rather than a single source. Parents remained the most credible authority for younger adolescents, but peers, teachers, and adolescent-friendly clinic staff were often the actors who translated that authority into action. For programme design, this means HPV communication for girls living with HIV should not target adolescents alone. It should deliberately include parents or guardians, use peer-support systems already present within adolescent HIV care, and strengthen the communication chain between schools, facilities, and households.

This study also found that the weak or uneven integration of HPV vaccination into routine HIV care. Many providers described counselling or referral rather than actual vaccination within the HIV care pathway, and some said HPV vaccination was not yet treated as a standard element of adolescent HIV services. This is consistent with the recent Mulago HIV-clinic study by Nakibuuka et al., which reported low vaccination uptake among adolescent girls living with HIV and noted that eligible girls were referred from the HIV clinic to other service points for vaccination [43]. It is also consistent with the Mulago completion study by Patrick et al., where completion was influenced by static and outreach service organisation rather than by a seamlessly integrated pathway inside HIV care [25].

The present study advances this literature by showing how weak integration actually feels in practice. For some participants, HPV vaccination existed as information in one unit, screening in another, and vaccination in yet another service point. For others, HPV was not part of the routine mental checklist of HIV care even though girls living with HIV were recognised as a priority group. This does not necessarily mean that every HIV clinic must directly stock and deliver HPV vaccine. The more practical implication to fit current service-integration agendas is that HIV clinics should reliably perform the functions that lie within their remit: identify eligible girls, provide accurate counselling, document vaccination status, align care pathways, and ensure that referrals are completed rather than merely advised. This interpretation is more realistic than concluding that HIV clinics are simply lagging behind.

Another major finding of this study was that practical barriers in access, documentation, and follow-up played a major role in non-initiation and especially non-completion of HPV vaccination. Participants described transport barriers, stock-outs, weak documentation, limited use of cards and registers, fragmented referral pathways, and poor follow-up for subsequent doses. These findings closely mirror the Kampala evidence from Mulago, where timely completion of the second HPV dose remained low and where improved social mobilisation, outreach, static vaccination approaches, and better education of eligible girls were recommended to strengthen completion [25]. They also resonate with findings from northern Uganda, where mobility between doses, school absenteeism, and weak follow-up disrupted completion [33]. In western Uganda, distance to the facility was significantly associated with uptake, again underscoring that service access remains a structural barrier even when the vaccine itself is provided free of charge [43].

Our study adds an important HIV-specific layer to these operational problems. Several key informants described a mismatch between ART appointment schedules and vaccine schedules, and some explicitly recommended aligning vaccination visits with routine HIV visits or embedding reminders into clinical systems.

#### Suggestions to improve HPV vaccine uptake and completion among adolescent girls living with HIV.

A number of adolescents and key informants suggested several strategies to improve HPV vaccination uptake among adolescents living with HIV. One of the most frequently emphasized was the availability of the HPV vaccine at the HIV drug refill sites for people living with HIV. Providing HPV vaccination at HIV treatment centers is critical, as most adolescents living with HIV adhere well to their hospital appointments and trust the healthcare workers at these facilities [36]. This approach would greatly reduce barriers related to access and improve HPV vaccination uptake. Participants proposed need for more intensive counselling, repeated school and community sensitization, stronger parent communication, more reliable vaccine availability, use of peers and VHTs, and deliberate reminder systems. Additionally, we propose contextually adapted community sensitization and stronger information systems, improved social mobilization, outreach, and education for eligible girls. Counselling from community members and community outreach platforms have the potential to support uptake among girls living with HIV.

Another key suggestion was increasing awareness and sensitization about HPV, HPV-related diseases, and the importance of vaccination among adolescents, their parents, healthcare workers, and the community at large [36]. Improved awareness would empower adolescents to seek out HPV vaccination and ensure timely completion of the recommended doses.

Consistent with findings from other studies [30,33,38,41], additional suggestions included ensuring continuous availability of vaccines at all facilities, increasing the number of trained healthcare workers to administer vaccines, strengthening school-based delivery programmes through engagement with school authorities, and integrating HPV vaccination into existing routine childhood immunisation and other adolescent health programmes. Enhancing school-based delivery remains a promising strategy, as it targets adolescents in settings where they spend most of their time and supports timely vaccination. This, however, must be adapted for adolescents living with HIV to ensure adherence to the recommended dosing schedule. A potential challenge is stigma, as adolescents may face questions from their peers if they are required to receive additional doses beyond what is provided to others. Reducing stigma is multi-faceted and requires input from different players/actors. Language use, use of mass media in destigmatizing HIV and HPV vaccines, health education on the disease and vaccine and drivers of stigma and fear and confidence building initiatives is critical in addressing the problem of stigma

Other proposed strategies included adopting a provider-initiated approach to HPV vaccination, given the low awareness among adolescents, and integrating HPV vaccination prompts into hospital information system, similar to existing systems for tuberculosis (TB) screening, so that patient records cannot be completed without documenting HPV vaccination status [36]. Provider recommendation has been shown to strongly influence vaccine uptake, as demonstrated by successful cervical cancer screening programmes among women living with HIV at almost all HIV treatment centers.

Some adolescents suggested mandatory vaccination policies and extending the vaccination to people’s homes and communities. While these approaches may increase coverage, they could face resistance if awareness is not accompanied by adequate community sensitization and education to foster understanding of the need for vaccination and encourage voluntary uptake. In this study, qualitative sample was designed to provide explanatory depth rather than statistical representativeness, and some beliefs captured in interviews reflect participant understanding rather than biomedical fact. However, those beliefs remain analytically important because they shape behaviour and help explain why routine health messages do not always translate into vaccine acceptance.

### Strengths of the study

This study provides a blueprint for using HIV treatment centers as routine or outreach vaccination sites targeting HIV-infected adolescents who would otherwise miss out on HPV vaccination. It clearly shows that focused vaccination strategies and structured follow-up programmes can significantly improve HPV vaccine uptake and completion rates. What our study contributes beyond earlier reports is a clearer articulation of how our proposed solutions should be configured for adolescent girls living with HIV. The HPV vaccination service improvement should work through structures these girls already use, e.g., adolescent clinic days, youth-friendly corners, peer supporters, caregiver counselling, refill visits, and HIV follow-up systems rather than alongside them. This study shows how those barriers interact within a Kampala Metropolitan service environment where HIV care, school-based vaccination, referral pathways, caregiver influence, and follow-up systems intersect. That makes the findings especially relevant for programme redesign in urban and peri-urban HIV care settings.

A second contribution is that the study highlights the interaction between informational barriers and organizational barriers. In much of the earlier literature, as referenced above, low uptake is discussed mainly in terms of poor knowledge, negative perceptions, or parental refusal [9,25,33–35,43]. Our findings confirm the importance of those factors, but also show that even motivated girls and caregivers may fail to initiate or complete vaccination when services are fragmented, documentation is weak, schedules are not aligned, or stock-outs interrupt trust.

### Limitations

Interruptions owing to shift of US government policy on funding HIV care and treatment programs through USAID interrupted the execution and conduct of this study. It remains to be seen how governments effectively navigate the funding gaps and how such changes will affect immunisation as a critical child survival and public health promotion strategy. Another limitation is that the parents/care givers were not included in the FGDs especially given their influence as highlighted by the adolescents and some key informants. The reasons listed under Table 3 were from open ended questions that were not categorized into percentages. Other limitations include: purposive FGD sampling, a single-city urban setting for the study, exclusion of adolescents already vaccinated which prevented comparison of completion motivators, no long-term follow-up beyond dose 2, and the absence of inferential statistics, and the fact that the follow up component did not include an improvement framework to define the process and outcome measures with denominators, fidelity assessment of the supportive supervision and webinar components, and a time-trend or run chart of dose one and dose two uptake across the study period.

## Conclusions

In these two urban HIV care and treatment centres, vaccination against HPV among eligible adolescents is way lower than recommended 90 percent. Adolescent girls living with HIV demonstrated low levels of knowledge about the HPV vaccine and poor access to vaccination services, despite being receptive to vaccination. This study showed that HPV vaccine uptake can be substantially improved within HIV care and treatment centers by reducing barriers to HPV vaccination uptake when the vaccines are made readily available and accessible, either in existing HIV care and treatment centres or in the new integrated care centres in areas where government programs have moved to implement HIV care integration into mainstream medical services. This study showed that stronger service models for girls living with HIV will depend on synchronizing vaccine follow-up with HIV appointments where feasible, documenting vaccination status within adolescent HIV records, and ensuring that girls are not lost between counselling, referral, vaccination, and follow-up.

## Recommendations

The Ministry of Health should introduce routine HPV vaccination clinics or services within HIV care and treatment programmes that operate independently of mainstream public health facilities. These programmes should ensure a consistent supply of vaccines, provide training for healthcare workers on HPV vaccination, strengthen social mobilization and community education, promote demand creation, and implement outreach services targeting unreached adolescents through tailored HPV vaccination initiatives. Improving HPV vaccination among adolescent girls living with HIV will require more than simply making vaccines available. It will require accurate and repeated counselling for girls and caregivers, explicit explanation of why girls living with HIV remain a priority group for two to three doses of HPV vaccine, deliberate engagement of parents as trusted decision-makers, stronger use of peer and school platforms, better integration of vaccination checks into adolescent HIV care, and practical systems for documentation and follow-up. Where the HIV clinics cannot become stand-alone vaccination centres, they should act as reliable entry points for identifying eligible girls, correcting misinformation, linking them promptly to vaccination, and ensuring they are not lost between counselling, vaccination, and follow-up.

## Supporting information

S1 File Data collection tools for girls and caregivers. (DOCX)

S2 File Additional data collection (and FGD) tools. (DOCX)
