# Supplementary material for: Strengthened HPV vaccination among adolescents living with HIV, – Findings of a Pilot QI project in Kampala Metropolitan, Uganda
Source: PLoS One. 2026 Aug 31;21(8):e0356686. doi: 10.1371/journal.pone.0356686 (PMC13528983; doi:10.1371/journal.pone.0356686)
Supplement: Supplementary file 1 [file pone.0356686.s001.docx]

**Appendix 2: Data Collection Tools for Girls and Caregivers**

**Understanding barriers and Strengthening Services for HPV vaccination among adolescents living with HIV in Kampala Metropolitan area.**

Study No: ……………	Date of Evaluation: ….... /…../………..

Day/Month/Year

Date enrolled in clinic ….../….../……….

**Socio-Demographic characteristics**

Name: …………………………………………………………

Age: ……………. Date of Birth: …………………………….

Address: village ………………………. District ……………………………

Telephone contact: ………………………………….

Weight……………………………... Height……………………………….

Currently in school	a) Yes	b) No

Class: …………….

Tribe ………………………………………….

Religion ………………………………………

Caregiver	a) Both mother and father

b) Mother alone

c) Father alone

d) Grandparents

e) Other ………………………….

Date of diagnosis of HIV infection………………………………………….

Age at diagnosis of HIV infection ………………………………………….

Do you know about your HIV status?

1) Yes……………….

2) No…………………….

If yes: who told you about your status?.......................................................

At what age were you when you first got to know about your status? (In years) …………….

What HAART regimen are you on?...............................................

What was your last CD4 count?

a) Count………

b) Date……

What was your last viral load?

a) Count…….
b) Date………

Do you know about the human papilloma virus (HPV)?

a) Yes
b) No

What is your view about the following regarding HPV? ………………………………

a) HPV is a virus; Yes…… No ………
b) HPV is sexually transmitted; Yes……………. No………………
c) HPV can cause cervical cancer; Yes…………. No ……………
d) Is there a relationship between HPV and HIV; Yes…… No…………
e) HPV occurs in girls alone; Yes…………. No………

Have you heard of cervical cancer?

a) Yes
b) No

What do you think causes cervical cancer? …………………………….

Have you heard about HPV vaccine?

a) Yes
b) No

How did you get to know about the HPV vaccine?

a) Health facility

b) Media

c) School

d) Friends

e) Other

What is the HPV vaccine used for?................

At what age should you receive the HPV vaccine?..............

How many doses are you supposed to receive?............

What is the dosing interval between the different doses of HPV vaccine?...........

Have you completed the HPV vaccination?...................

a) Yes
b) No

How many doses have you received? ……………………………………..

Why haven’t you completed the HPV vaccination? (Tick applicable options)

a) I did not know that I needed to
b) I do not find it safe
c) I don’t think it works
d) My care giver discouraged me from it
e) No one offered it to me
f) The vaccine is painful
g) The vaccine is harmful
h) My health facility did not have the vaccine
i) Others

Why haven’t you started HPV vaccination?

a) I did not know that I needed to
b) I do not find it safe
c) I don’t think it works
d) My care giver discouraged me from it
e) No one offered it to me
f) The vaccine is painful
g) The vaccine is harmful
h) My health facility did not have the vaccine
i) Others

When did you get the doses you have received?

a) Date of first dose received: ……………………………
b) Date of second dose received: ……………………………
c) Date of third dose received……………….

Do you have any documentation that shows you received the vaccine?

1. Yes
2. No

Interviewer reviewed card

1. Card reviewed up to date
2. Card reviewed not up to date
3. Card not reviewed

What motivated you to start or complete HPV vaccination? (Tick applicable options)

a) Recommendation by care taker
b) Recommendation health care giver
c) Community outreach
d) Peers were getting
e) School program
f) TV or radio show
g) Distance to health facility
h) Availability of vaccine at the health facility where ART is received
i) Other

Are you sexually active?

1) Yes

2) No

If yes; how old were you when you first had sex?

How many sexual partners do you have?

Do you have children?

a) Yes
b) No

If yes;

a) How many children do you have? ……………….
b) How old were you when you had your first child? .................

Thank you for your participation.
