# Supplementary material for: Strengthened HPV vaccination among adolescents living with HIV, – Findings of a Pilot QI project in Kampala Metropolitan, Uganda
Source: PLoS One. 2026 Aug 31;21(8):e0356686. doi: 10.1371/journal.pone.0356686 (PMC13528983; doi:10.1371/journal.pone.0356686)
Supplement: Supplementary file 2 [file pone.0356686.s002.docx]

**Appendix 3: Additional data collection (and FGD) tools**

Good morning/ afternoon,

Thank you all for agreeing to participate in this study.

My name is ……………………………. and this is my colleague …………………………. This study is being carried out by Dr. Ombeva Malande and team from Makerere University, Department of Paediatrics and Child Health alongside other collaborators.

We are conducting several discussions to determine the factors associated with HPV vaccine uptake among girls 10-19 years living with HIV. Your opinions are very important; please feel free to share your concerns and ideas about the topic that comes up in today’s discussion. Your responses will serve as basis to improve HPV vaccine completion levels in the country.

The discussion will take between 30 – 45 minutes. All information you provide here will be confidential. There are no wrong answers and you do not have to agree with what others say during the discussion. We ask for permission to audio record this session because we do not want to miss any of your comments. There will be no names attached to your views.

1. What do you know about the Human Papilloma Virus? *(Probe for HPV transmission, what HPV causes, HPV prevention if not mentioned)*
2. What do you know about cervical cancer? (*Probe for knowledge about cause, risk factors and prevention)*
3. Have you heard of Human Papilloma Virus vaccine? *if yes what are your sources?*
4. Why is vaccination against Human Papilloma Virus done? (*Probe for importance of vaccine if not mentioned.)*
5. What do you know about the human papilloma virus vaccine? (*Probe about the number of doses, how far apart they are given, and the age at which it is given)*
6. Are there any adolescents who haven’t started the HPV vaccination? What are some of the reasons?

*Probe for barriers to HPV vaccine completion – age of adolescent, parental consent, fear of side effects, unaware of dose schedule, inadequate information about vaccine, sexual activity/risky sexual behaviour, HCW recommendation, availability of vaccine at the facility, boarding vs day school, subsequent school visits were not done*

7. What would you say encourages people to start the HPV vaccine?

*Probe on the motivators of HPV vaccination – HCW recommendation, gender of HCW, awareness of HPV vaccine benefits, parental wishes or peer influence, sexual activity, perceived risk of HPV infection*

8. What do you think should be done to improve uptake and completion of HPV vaccine in Uganda?
9. Do you have any other comment or questions you would like us to talk about in relation to HPV vaccine?

Thank you for your participation.
